# PReS-FINAL-2266: A rare cause for childhood uveitis: TINU (tubulointerstitial nephritis and uveitis) syndrome

**DOI:** 10.1186/1546-0096-11-S2-P256

**Published:** 2013-12-05

**Authors:** K Barut, T Rızayev, A Yurt, I Tugal-Tutkun, S Çalışkan, N Canpolat, L Sever, O Kasapcopur

**Affiliations:** 1 Pediatric Rheumatology, Istanbul University, Cerrahpasa Medical Faculty, Turkey; 2 Pediatric Nephrology, Istanbul University, Cerrahpasa Medical Faculty, Turkey; 3 Ophthalmology, Istanbul University, Istanbul Medical Faculty, Istanbul, Turkey

## Introduction

It is very difficult to determine the etiology of isolated uveitis during childhood. If a patient presenting with uveitis has an associated acute interstitial nephritis; tubulointerstitial nephritis and uveitis (TINU) syndrome should be considered in the differential diagnosis.

## Objectives

In this case report, a 15 year old girl presenting with uveitis associated with glucosuria and an increase in creatinine, and who were diagnosed as TINU syndrome will be discussed.

## Methods: case report

15-year-old female admitted with 20 day duration of extreme fatigue, loss of appetite, weight loss and redness in eyes and decreased vision. Ophthalmologic examination suggested bilateral anterior uveitis and right eye anterior granulomatosis uveitis. We found mild renal insufficiency; serum urea was 76 mg/dl, creatinine level was 1,2 mg/dl both of which were elevated. The patient's erythrocyte sedimentation rate was 112 mm/hour, complete blood count and other biochemical parameters were normal. Antinuclear antibodies were positive in speckled pattern. Urinalysis showed low urine density, normoglycemic glycosuria and nonnephrotic proteinuria and we found high urinary β-2 microglobulin levels (45.3 mg/L, normal values: 0.02-0.25 mg/L). A renal biopsy was performed. The biopsy specimen showed dense lymphocytes, plasmocytes and variable eosinophiles in the interstitium, tubulitis in the tubule, focal debris and hyaline cylinders in the tubule. Glomerular structures were preserved. These findings were compatible with acute tubulointerstitial nephritis. With all of these findings, the patient was diagnosed as TINU syndrome. The patient received 2 mg/kg of prednisone for one month. Her kidney function normalized after prednisone therapy. Uveitis responded to systemic and local corticosteroid treatment. At her follow-up, vision was completely resolved.

## Conclusion

In a patient with uveitis, urinalysis should be done during investigation of the underlying etiologic causes. Pathologic findings in urinalysis should remind a probable diagnosis of TINU syndrome. TINU syndrome in children responds very well to systemic and local corticosteroid treatment.

## Disclosure of interest

None declared.

